# Lipopolysaccharide-induced immune stress negatively regulates broiler chicken growth *via* the COX-2-PGE_2_-EP4 signaling pathway

**DOI:** 10.3389/fimmu.2023.1193798

**Published:** 2023-05-03

**Authors:** Kexin Liu, Wenrui Zhen, Dongying Bai, Haiqiu Tan, Xianglong He, Yuqian Li, Yanhao Liu, Yi Zhang, Koichi Ito, Bingkun Zhang, Yanbo Ma

**Affiliations:** ^1^ Department of Animal Physiology, College of Animal Science and Technology, Henan University of Science and Technology, Luoyang, China; ^2^ Longmen Laboratory, Science & Technology Innovation Center for Completed Set Equipment, Luoyang, China; ^3^ Henan International Joint Laboratory of Animal Welfare and Health Breeding, College of Animal Science and Technology, Henan University of Science and Technology, Luoyang, China; ^4^ Department of Food and Physiological Models, Graduate School of Agricultural and Life Sciences, The University of Tokyo, Ibaraki, Japan; ^5^ State Key Laboratory of Animal Nutrition, Department of Animal Nutrition and Feed Science, College of Animal Science and Technology, China Agricultural University, Beijing, China

**Keywords:** immune stress, hypothalamus, COX-2, growth, broilers

## Abstract

**Aims:**

Immune stress in broiler chickens is characterized by the development of persistent pro-inflammatory responses that contribute to degradation of production performance. However, the underlying mechanisms that cause growth inhibition of broilers with immune stress are not well defined.

**Methods:**

A total of 252 1-day-old Arbor Acres(AA) broilers were randomly allocated to three groups with six replicates per group and 14 broilers per replicate. The three groups comprised a saline control group, an Lipopolysaccharide (LPS) (immune stress) group, and an LPS and celecoxib group corresponding to an immune stress group treated with a selective COX-2 inhibitor. Birds in LPS group and saline group were intraperitoneally injected with the same amount of LPS or saline from 14d of age for 3 consecutive days. And birds in the LPS and celecoxib group were given a single intraperitoneal injection of celecoxib 15 min prior to LPS injection at 14 d of age.

**Results:**

The feed intake and body weight gain of broilers were suppressed in response to immune stress induced by LPS which is an intrinsic component of the outer membrane of Gram-negative bacteria. Cyclooxygenase-2 (COX-2), a key enzyme that mediates prostaglandin synthesis, was up-regulated through MAPK-NF-κB pathways in activated microglia cells in broilers exposed to LPS. Subsequently, the binding of prostaglandin E2 (PGE2) to the EP4 receptor maintained the activation of microglia and promoted the secretion of cytokines interleukin-1β and interleukin-8, and chemokines CX3CL1 and CCL4. In addition, the expression of appetite suppressor proopiomelanocortin protein was increased and the levels of growth hormone-releasing hormone were reduced in the hypothalamus. These effects resulted in decreased expression of insulin-like growth factor in the serum of stressed broilers. In contrast, inhibition of COX-2 normalized pro-inflammatory cytokine levels and promoted the expression of Neuropeptide Y and growth hormone-releasing hormone in the hypothalamus which improved the growth performance of stressed broilers. Transcriptomic analysis of the hypothalamus of stressed broilers showed that inhibition of COX-2 activity significantly down-regulated the expression of the TLR1B, IRF7, LY96, MAP3K8, CX3CL1, and CCL4 genes in the MAPK-NF-κB signaling pathway.

**Conclusion:**

This study provides new evidence that immune stress mediates growth suppression in broilers by activating the COX-2-PGE2-EP4 signaling axis. Moreover, growth inhibition is reversed by inhibiting the activity of COX-2 under stressed conditions. These observations suggest new approaches for promoting the health of broiler chickens reared in intensive conditions.

## 1 Introduction

Broiler chickens grown under intensive breeding conditions commonly are infected by diverse pathogenic and nonpathogenic microorganisms. These infections frequently induce immune stress in broilers. Immune stress may cause abnormal changes in the behavior and metabolism of broilers which lead to the decline of immunity and disease resistance and ultimately result in growth inhibition. Thus, immune stress is a key factor that hinders broilers from achieving optimal rates of feed and ideal growth and thereby causes significant damage to the poultry breeding industry (1–3). Immune-stressed broilers produce increased levels of inflammatory cytokines which induce fever and anorexia (4, 5). Moreover, these inflammatory cytokines activate the hypothalamic-pituitary-adrenal (HPA) axis and stimulate the adrenal gland to secrete corticosterone (CORT) which accelerates the oxidative decomposition of muscle (6, 7). Despite these intriguing observations, the mechanism by which growth is restrained in immune-stressed broilers has not been elucidated fully.

Expression of cyclooxygenase-2 (COX-2), a key enzyme in the biosynthesis of prostaglandins (PGs), is enhanced in the hypothalamus following stimulation by lipopolysaccharide (LPS) (8, 9). LPS is an intrinsic component of the outer membrane of Gram-negative bacteria and is a potent activator of the immune system. Nevertheless, LPS-induced decrease in feed intake of stressed broilers was ameliorated significantly by treatment with the COX-2 inhibitors celecoxib or indomethacin (10), which suggests that immune stress-induced anorexia may be mediated by COX-2. PGs are inflammatory mediators that are elevated in the brain during inflammation and which modify key aspects of neuronal activity to evoke symptoms of sickness behavior (11). Prostaglandin H_2_, the end product of COX enzyme activity, is converted into diverse PGs, including PGD_2_, PGF_2α_, PGE_2_, PGI_2_, and thromboxane A2 (12), each of which exerts specific physiological effects. Interestingly, PGE_2_ was the most potent PG that induced anorexia by suppressing food intake in mice *via* the EP4 receptor. This receptor is one of four receptor subtypes for PGE_2_ (13, 14). However, the mechanism by which PGE_2_ regulates food intake in broilers remains to be explored. We previously demonstrated that the expression of COX-2 and the downstream mPGEs-1 gene that encodes microsomal prostaglandin E synthase-1, which is a terminal PGE2 synthase, promoted the excitability of hypothalamic corticotropin-releasing hormone nerve cells and activation of the HPA axis (15, 16). These data led us to test the potential involvement of COX-2 and its major downstream product PGE_2_ in growth inhibition of broilers under immune stress conditions. Therefore, in this study we constructed a broiler immune stress model by intraperitoneal injection of LPS to investigate stress-induced expression of the COX-2 signaling pathway and the cascade of PGE_2_ receptors (EP receptors) in the hypothalamus.

## 2 Materials and methods

### 2.1 Animals and treatment

Two hundred and fifty-two one day old Arbor Acres broiler chickens were purchased from Henan Quanda Poultry Breeding Company. Broilers were randomly allocated to three groups with six replicates per group and fourteen broilers per replicate. The three groups comprised a saline control group, an LPS (immune stress) group, and an LPS and celecoxib group corresponding to an immune stress group treated with a selective COX-2 inhibitor. The chickens had *ad libitum* access to basal diet and water in a temperature- and light-controlled facility with a light schedule of continuous light for the first three days, and then maintained 23L:1D for the remainder of the study. Chickens in the stress group at 14 d of age were injected intraperitoneally with LPS (0.5 mg/kg) from *Escherichia coli* serotype 055: B5 (Sigma, St. Louis, MO, USA) once a day for three days to induce the immune stress model. In parallel, chickens in the control group were injected with the same concentration of pyrogen-free NaCl solution (0.9%). Besides, chickens in the LPS and celecoxib group were given a single intraperitoneal injection of celecoxib (10 mg/kg; Sigma) diluted in DMSO (Sigma) 15 min prior to LPS injection at 14 d of age. Thus, chickens in all groups received two injections at 14 d of age at 15 min intervals. All experiments were performed according to animal ethics guidelines and approved protocols of the Animal Care and Use Committee of Henan University of Science and Technology.

### 2.2 Sample collection

Chickens from each replicate, a total of six chickens in each tested group were selected 2 h, 4 h, 24 h, and 72 h after the second intraperitoneal injection at 14 d. Blood samples were collected through wing vein puncture. Serum was separated by centrifugation at 3000 g for 30 min at 4°C and was frozen immediately at -80°C until subsequent analysis. Sampled birds were euthanized by cervical dislocation. The brains were removed quickly and the hypothalamus were excised, fresh-frozen in liquid nitrogen, and stored at -80°C for further analysis. The duodenum, jejunum, and ileum were collected for length measurements and the spleen, thymus, and bursa were enucleated to calculate the immune organ index 24 h and 72 h after the second intraperitoneal injection at 14 d.

### 2.3 Growth performance determination

At 14, 15, and 17 d corresponding to 0 h, 24 h, and 72 h after the second intraperitoneal injection at 14 d, chickens were weighed and the total feed consumption was recorded to calculate average daily gain, average daily feed intake, and feed conversion ratio.

### 2.4 Serum CORT, TNF-α, and IGF-concentration determination

Serum concentrations of CORT, TNF-α, and IGF-1 were determined through enzyme-linked immunosorbent assay (ELISA) using kits from the Nanjing Jiancheng Haihao Biotechnology Co., Ltd. (Nanjing, China) and procedures provided in the kit.

### 2.5 Sequence analysis

At 2h after the second intraperitoneal injection at 14 d, four hypothalamus samples were selected randomly from different treatment groups and were subjected to transcriptome sequencing (Nanjing Parsono Technology Co., Ltd., China). Following sample RNA extraction, purification and library construction, libraries were sequenced using next-generation sequencing based on the Illumina Hiseq sequencing platform after library quantification. Raw data were filtered and quality controlled and Q20, Q30, and GC content were calculated for valid data (clean data). The filtered clean data subsequently were aligned to the reference genome of chicken (*Gallus gallus*) using HISAT2 software to obtain alignment efficiency of the sequences and to map sequence information on the reference genome. Differential expression analysis was performed using DESeq2 software ([Log2 (Fold Change)] ≥ 1 and *p<0.05*). Genes that met these two conditions simultaneously were considered as genes that were differentially expressed significantly. Enrichment analysis of these genes was performed in Gene Ontology (GO) and Kyoto Encyclopedia of Genes and Genomes (KEGG) pathway using R script. The Fisher exact test combined with Bonferroni multiple test was used to control and calculate the false positive rate. A *p* < 0.05 was considered significant enrichment.

### 2.6 Real-time quantitative polymerase chain reaction

Trizol reagent (Invitrogen Inc., Carlsbad, CA, USA) was used to isolate total RNA from the hypothalamus. RNA quality was assessed by measuring the 260/280 ratio. The first strand cDNA synthesis kit (Toyobo, Osaka, Japan) was used for the production of cDNA. The target genes and HPRT loading control gene primers are shown in **Table 1**. Real-time quantitative polymerase chain reaction (qPCR) was performed with a real-time PCR kit (Toyobo) in a Roche LightCycle instrument (Shanghai, China). Data were analysed by the 2^−ΔΔCT^ method.

**Table 1:** Primers used for quantitative real-time PCR.

Target gene^1^	Primer	Sequence (5’–3’)
HPRT	Forward	CAGCCCCTGCATCGTGATTG
	Reverse	TTCACGTGCCAGTCTCTCTG
CD40	Forward	GCCTGGTGATGCTGTGAATT
	Reverse	TGCAGTGCCTTTCCTTTGTC
IKBKE	Forward	CCATTCTGGCCAACATCCTG
	Reverse	AGGAGTGGATGTAGATGCGG
GHRH	Forward	GGGGAGAAAAGGCAAAGCAA
	Reverse	GTGTTGTCCGCTTCCCTTTT
IL1β	Forward	TGGGCATCAAGGGCTACAAG
	Reverse	AGGCGGTAGAAGATGAAGCG
SST	Forward	CCAGACCAGCAGAGAGGAAA
	Reverse	ACAGAACAGAGCAGAGCACT
MAP3K14	Forward	CAATTACCCAGACAGCGAGC
	Reverse	TCAAGGCGTCCACCAAGTAT
MAP3K8	Forward	CAGATGTGCTCCTGTTTCCG
	Reverse	TTGAACTGTTCCACTGGCAC
MYD88	Forward	GATGATCCGTATGGGCATGG
	Reverse	CACGTTCCTGGCAAGACATC
NPY	Forward	GTGCTGACTTTCGCCTTGTC
	Reverse	ATCTCTGCCTGGTGATGAGG
POMC	Forward	AAAGAAGGATGGAGGCTCGT
	Reverse	TGTAGGCGCTTTTGACGATG
COX-2	Forward	CCGAATCGCAGCTGAATTCA
	Reverse	GAAAGGCCATGTTCCAGCAT
SOCS3	Forward	ACCCCAAACGCACCTACTAC
	Reverse	GTGCCCGTTGACAGTCTTAC
STAT1	Forward	CACCCAATGCCTGTCTTGAC
	Reverse	GAAAAGACTGTGCGTTCGGT
TLR1B	Forward	GAGGCGTAACGACACATCCT
	Reverse	TTTGGGACATCACTCCACGG
PGIS	Forward	GTGGCTGCTATCACCGATTC
	Reverse	GGTTCTCCTCATTGCCTTCC
PGDS	Forward	ACAGTGCGAGAAGAGGAACA
	Reverse	GTGCTCTTGGAGATCTGGGT
EP2	Forward	CTACACCTTCTCAGCTGCCT
	Reverse	TGAGGAAGAGGAGCAAGGTG
EP3	Forward	AATCATGCAAGGGGTTCTCG
	Reverse	GAGCAGCAGATAAACCCACG
EP4	Forward	CATGGGGCTCAGCAAATCAA
	Reverse	CAGGATGTTGCAGATCACCG
mPGEs-1	Forward	AGGCTCAGGAAGAAGGCATT
	Reverse	CACAGCTCCAAGGAAGAGGA

### 2.7 Statistical analysis

Data analysis was performed with SPSS statistical software (ver. 21.0 for Windows, SPSS Inc., Chicago, USA) by using a *t*-test followed by Duncan multiple comparison tests. Significance (*p*-value) was evaluated at 0.05.

## 3 Results

### 3.1 LPS-induced immune stress regulates the MAPK-NF-κB signaling pathway

**Figure 1:**
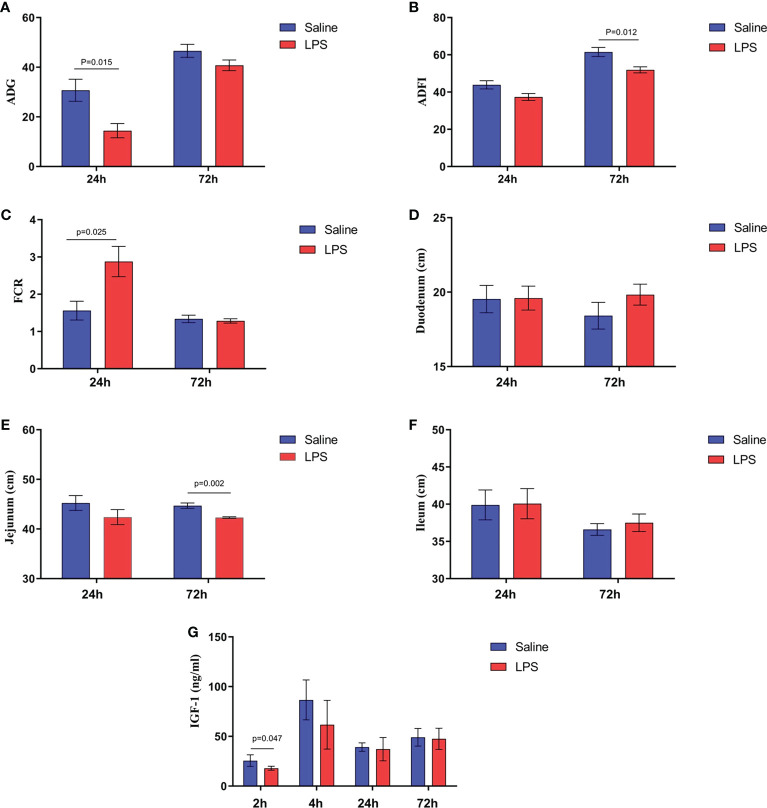
LPS-induced immune stress regulated the MAPK-NF-κB signaling pathway in broilers. **(A-C)** Production performance and length of **(D)** duodenum, **(E)** jejunum, and **(F)** ileum after the time from the first injection. **(G)** Serum levels of IGF-1 after the time from the first injection. Values are expressed as mean ± SEM. P < 0.05 indicates significant difference between groups.

**Figure 2:**
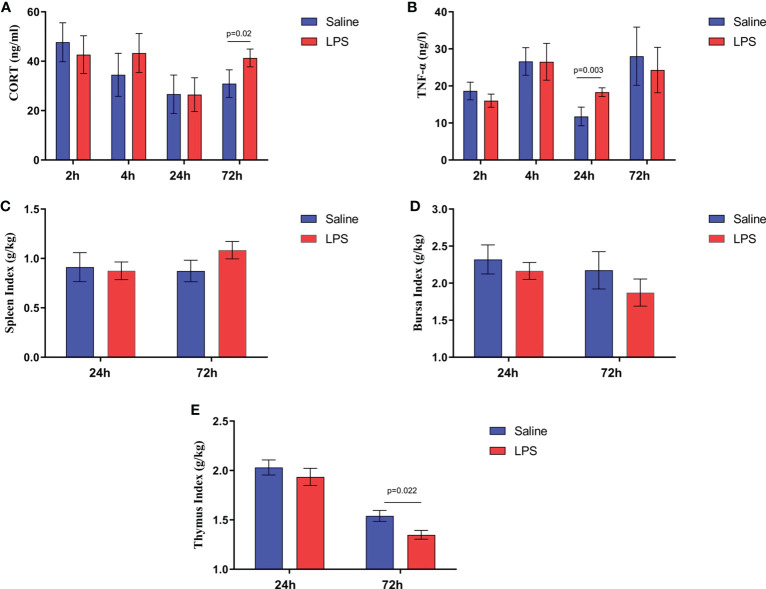
LPS-induced immune stress regulated the MAPK-NF-κB signaling pathway in broilers. Serum levels of **(A)** CORT and **(B)** TNF-α after the time from the first injection. Index of **(C)** spleen, **(D)** bursa, and **(E)** thymus after the time from the first injection. Values are expressed as mean ± SEM. P < 0.05 indicates significant difference between groups.

The impact of stress on the growth performance and immune function of broilers was examined using an LPS-induced stress model. We observed that the growth performance and immune function of animals in the stress group were suppressed significantly (*p* < 0.05) compared to the control group that was administered saline only. Specifically, LPS challenge reduced both the body weight gain 24 h after the first injection and the feed intake 72 h after injection (**Figures 1A, B** **)**, whereas the feed conversion ratio increased 24 h after the first LPS injection (*p* < 0.05) (**Figure 1C**). Moreover, the jejunum length was decreased significantly *(p* < 0.05) in animals in the LPS-treated group compared to the control group (**Figure 1E**). No significant changes were observed in duodenum and ileum length *(p* > 0.05) (**Figures 1D, F** **)**. IGF-1 levels in the serum of stressed broilers were down-regulated (*p* < 0.05) 2 h after the first LPS injection (**Figure 1G**). In addition, LPS challenge elevated concentrations of TNF-α and CORT in serum (*p* < 0.05) 24 h and 72 h, respectively, after the first LPS injection (**Figures 2A, B** **)**. The weight index of immune organs also may be used to evaluate the immune status of poultry (17). Accordingly, LPS treatment significantly inhibited the thymus index in broilers (**Figure 2E**). No significant changes were observed on the spleen index and bursa index of fabricius *(p* > 0.05) (**Figures 2C, D** **)**.

**Figure 3:**
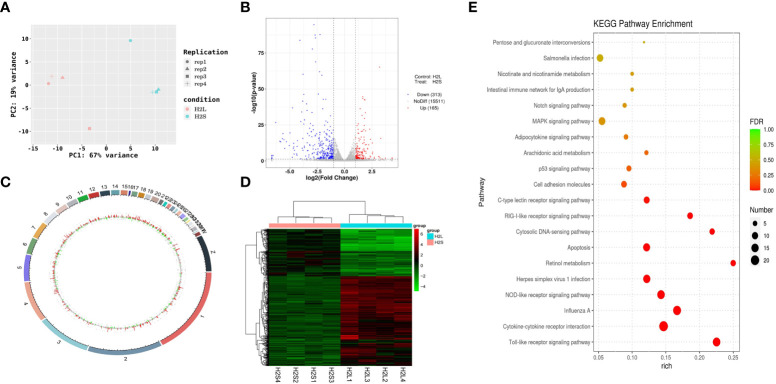
LPS-induced immune stress regulated the MAPK-NF-κB signaling pathway in broilers. H2S, saline group; H2L, LPS group; **(A)** Principal component analysis of hypothalamus transcriptomics 2 h after the first injection. **(B)** Volcano plot of hypothalamus collected from stressed broilers compared to control broilers 2 h after the first injection. Red and blue dots indicate genes that are absolute-value [log2(FC)] ≥ 1 and FDR < 0.05. **(C)** Circos heatmap of differentially expressed genes in stressed broilers and control broilers 2 h after the first injection. **(D)** Heatmap showed the 478 differentially expressed genes between stressed broilers and control broilers 2 h after the first injection. **(E)** Scatterplot of enrichment analysis of KEGG pathway of differentially expressed genes in stressed broilers and control broilers 2 h after the first injection.

**Figure 4:**
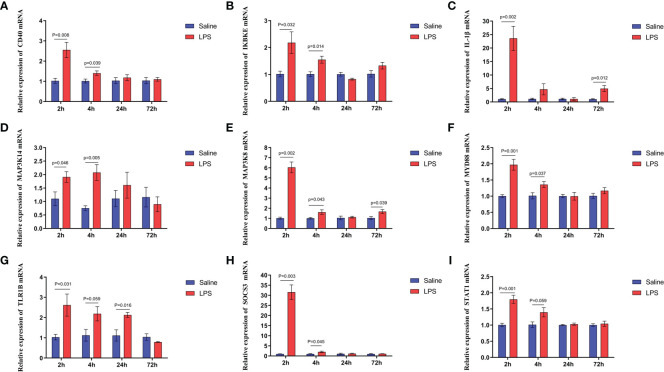
LPS-induced immune stress regulated the MAPK-NF-κB signaling pathway in broilers. Relative mRNA expression of **(A)** CD40, **(B)** IKBKE, **(C)** IL-1β, **(D)** MAP3K14, **(E)** MAP3K8, **(F)** MYD88, **(G)** TLR1B, **(H)** SOCS3, and **(I)** STAT1 in hypothalamus. Values are expressed as mean ± SEM. P < 0.05 indicates significant difference between groups.

**Figure 5:**
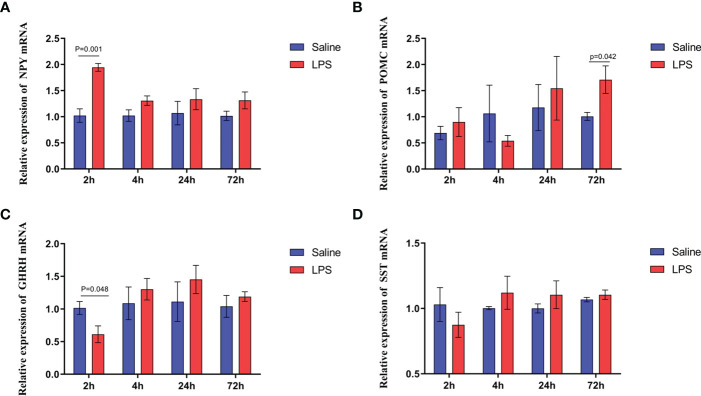
LPS-induced immune stress regulated the MAPK-NF-κB signaling pathway in broilers. Relative mRNA expression of **(A)** NPY, **(B)** POMC, **(C)** GHRH, and **(D)** SST in hypothalamus. Values are expressed as mean ± SEM. P < 0.05 indicates significant difference between groups.

The mechanism by which immune stress inhibited growth and immunity in broilers was assessed further by hypothalamus transcriptome analysis (**Figure 3**). Four hypothalami from each group were selected randomly for study. Three-hundred and thirteen genes were up-regulated and 165 genes were down-regulated in the hypothalamus of the LPS-stressed group compared with the control group (*p <*0.05) (**Figure 3B**). Heatmap analysis revealed that gene expression in the samples within each group was consistent, but that differential gene expression changes occurred between the LPS-treated and control groups (**Figure 3D**). Thus, the repeatability and intragroup consistency were high in these experiments. The KEGG pathway analysis revealed that the differentially expressed genes in the hypothalamus were associated with 14 significant gene pathways (**Figure 3E**). Eight pathways related to growth and immunity of chickens were examined further: Toll-like receptor signaling pathway, cytokine-cytokine receptor interaction, NOD-like receptor signaling pathway, RIG-I-like receptor signaling pathway, cytosolic DNA-sensing pathway, C-type lectin receptor signaling pathway, adipocytokine signaling pathway, and arachidonic acid metabolism. Thirteen genes were selected for validation. Compared with the control group, the mRNA levels of the genes for IL-1β, CD40, STAT1, TLR1B, MYD88, IKBKE, MAP3K8, MAP3K14, SOCS3, NPY, and POMC were up-regulated significantly and the gene for growth hormone–releasing hormone (GHRH) was down-regulated *(p* < 0.05) (**Figures 4A-I** **,** **5A-C**), whereas the expression level of the somatostatin (SST) gene was unchanged in the stress group compared to the saline only group *(p* > 0.05) (**Figure 5D**). These observations are consistent with the trends described above for transcriptome analysis of the hypothalamus.

The preceding data demonstrated that LPS-induced immune stress upregulated the expression of TLR1B and then activated the Toll-like receptor signaling pathway (*p* < 0.05). The Toll-like receptor TLR1B protein binds to myeloid differentiation factor 88 (MyD88) (18, 19) which leads to the activation of the TNFRSF6B and IRF7 genes which leads to the activation of the MAPK and NF-κB signaling pathways *via* up-regulation of expression of the MAP3K8, NFKBIA, IKBKE, and MAP3K14 genes. Subsequently, NF-κB rapidly translocates to the nucleus and binds to specific DNA response elements to promote gene transcription and MAPK pathway also regulates the downstream activator of the STAT1 factor to promote the transcription and production of pro-inflammatory factors (20–22), which results in the increased secretion of cytokines IL1β and IL-8 and chemokines CX3CL1 and CCL4 (**Table S1**).

### 3.2 LPS-induced immune stress upregulates the COX-2-PGE2-EP4 signaling pathway

**Figure 6:**
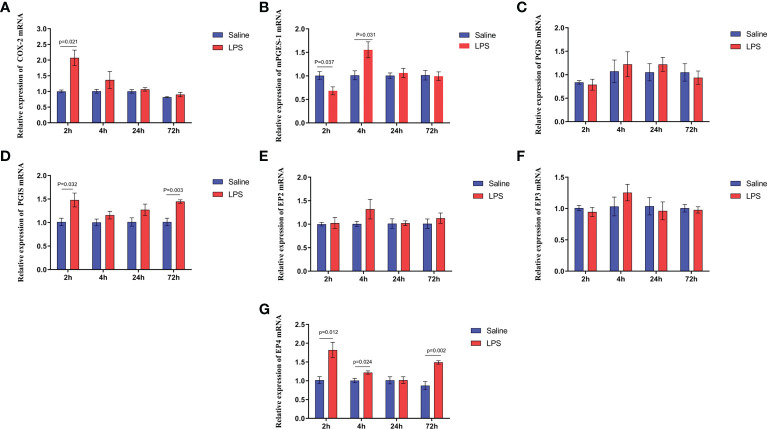
LPS-induced immune stress upregulates the COX-2-PGE2-EP4 signaling pathway in broilers. Relative mRNA expression of **(A)** COX-2, **(B)** mPGEs-1, **(C)** PGDS, **(D)** PGIS, **(E)** EP2, **(F)** EP3 and **(G)** EP4 in hypothalamus after the time from the first injection. Values are expressed as mean ± SEM. P < 0.05 indicates significant difference between groups.

A significant increase in the expression of COX-2 and PGE_2_ synthetase in the hypothalamus isolated from immune stressed broilers occurred compared with broilers from the control group *(p* < 0.05) (**Figures 6A, B** **)**, and LPS increased the level of PGI_2_ synthetase *(p* < 0.05), showing no effect on the PGD_2_ synthetase *(p >*0.05) (**Figures 6C, D** **)**. The expression of genes that encode four EP receptors in the hypothalamus after LPS challenge was measured, the mRNA levels of the EP4 receptor in the hippocampus increased significantly after LPS injection (*p* < 0.05), whereas LPS administration did not affect the expression of the genes for the EP2 and EP3 receptors (*p* > 0.05) (**Figures 6E-G**). These data suggest that COX-PGE_2_-EP4 signaling in the hypothalamus may be activated during growth inhibition of broilers under immune stress.

### 3.3 COX-2 inhibition attenuates LPS-induced growth reduction and inflammation

**Figure 7:**
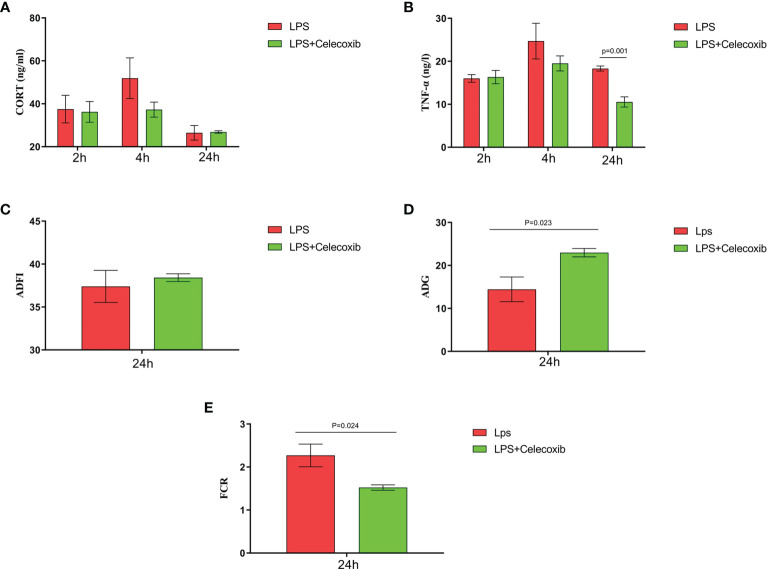
COX-2 inhibitor celecoxib attenuated LPS-induced growth inhibition and inflammation in broilers. Serum levels of **(A)** CORT and **(B)** TNF-α and **(C-E)** production performance after the time from the first injection. Values are expressed as mean ± SEM. P < 0.05 indicates significant difference between groups.

**Figure 8:**
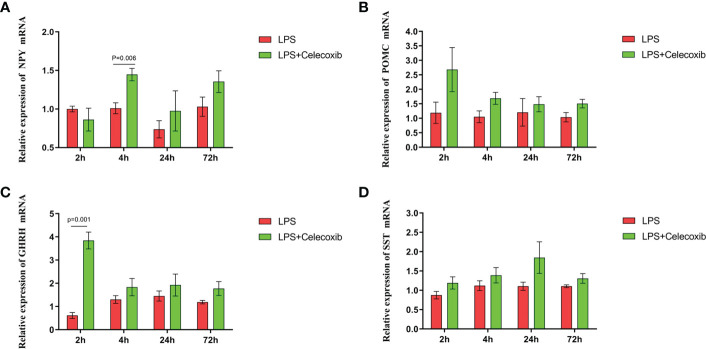
COX-2 inhibitor celecoxib attenuated LPS-induced growth inhibition and inflammation in broilers. Relative mRNA expression of **(A)** NPY, **(B)** POMC, **(C)** GHRH and **(D)** SST in hypothalamus. Values are expressed as mean ± SEM. P < 0.05 indicates significant difference between groups.

**Figure 9:**
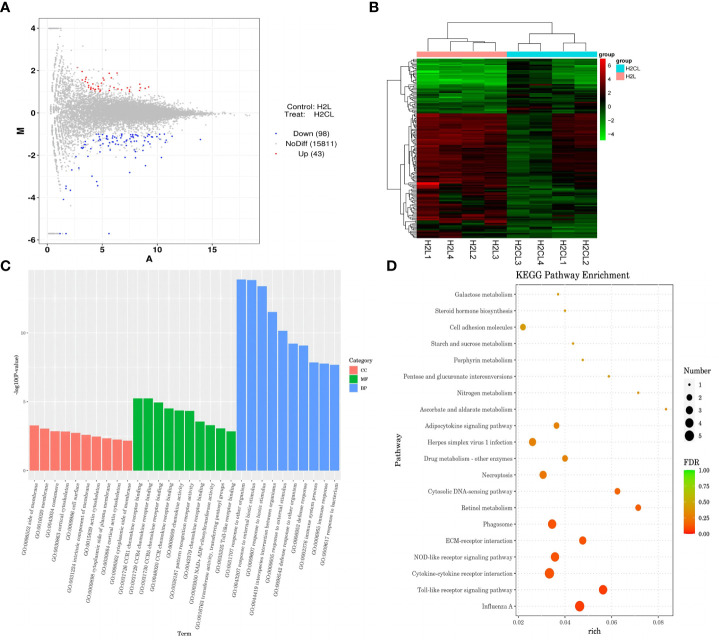
COX-2 inhibitor celecoxib attenuated LPS-induced growth inhibition and inflammation in broilers. H2L, LPS group; H2N, LPS + celecoxib group. **(A)** Plot of hypothalamus collected from the LPS with celecoxib group compared to LPS group 2 h after the first injection. Red and blue dots indicate genes that are absolute-value [log2(FC)] ≥ 1 and FDR < 0.05. **(B)** Heatmap showed 376 genes that are differentially expressed in the LPS with celecoxib group and LPS group 2 h after the first injection. **(C)** Top 10 signaling pathways from the GO classification and enrichment analysis comparing the LPS with celecoxib group and LPS group. **(D)** Scatterplot of enrichment analysis of KEGG pathway of differentially expressed genes comparing the LPS with celecoxib group and LPS group.

The relationship between COX-2-related signaling and the MAPK-NF-κB signaling pathway in immune stressed broilers was explored further using the brain-penetrant COX-2 inhibitor celecoxib (**Figures 7A–E**). The compound was supplied by intraperitoneal injection prior to administration of LPS. TNF-α levels in the blood (**Figure 7B**) and proinflammatory factors in the hypothalamus were down-regulated after celecoxib treatment compared with broilers that were injected with LPS only (*p* < 0.05). Celecoxib also reduced significantly (*p* < 0.05) the levels of CD40 which is a lysosomal protein associated with microglial activation (23) (**Table S2**). Moreover, the growth performance of stressed broilers was improved after inhibition of COX-2 which was reflected in increased body weight gain and decreased feed conversion ratio of broilers in the celecoxib treated group 24 h after the primary LPS injection (*p* < 0.05) (**Figures 7C-E**). We subsequently examined the expression levels of the genes for NPY, POMC, GHRH, and SST in the hypothalamus. Celecoxib pretreatment significantly increased GHRH expression at 2 h and NPY expression at 4 h after the first LPS injection compared with the stress group without celecoxib (*p* < 0.05) (**Figures 8A, C** **)**. However, the expression level of POMC and SST did not differ between the two groups (*p* > 0.05) (**Figures 8B, D** **)**. Moreover, transcriptomic analysis of the hypothalamus showed that celecoxib markedly downregulated the cytokine-cytokine receptor interaction and the Toll-like receptor signaling pathway (**Figures 9A-D**) and also suppressed the expression of the TLR1B, TNFRSF6B, IRF7, LY96, MAP3K8, CX3CL1, and CCL4 genes compared with LPS-challenged broilers without celecoxib treatment (**Table S2**). These results suggest that the inhibitory effect of LPS-induced stress on broiler immunity and growth was mediated partially by COX-2.

## 4 Discussion

LPS-induced immunological stress activates persistent pro-inflammatory responses that contribute to degradation of production performance in broilers (24–26). However, the molecular mechanisms that underpin growth inhibition caused by immunological stress remain elusive. The purpose of this study was to investigate the mechanism by which immunological stress restrains broiler productivity. We found that the performance of broilers that were stressed by LPS challenge was reduced which was reflected in a decrease of body weight gain 24 h after the first LPS injection as well as a reduction in feed intake and in the jejunum index, particularly 72 h after LPS administration. The HPA axis is activated when broilers are exposed to stressors which results in a sharp rise in CORT concentrations (27). Thus, CORT levels provide a reliable indicator of chicken stress status. In agreement, we found that LPS challenge increased CORT concentrations in the serum of broilers which indicates that the broiler immune stress model was valid. In addition, we observed that LPS-induced stress elevated TNF-α levels in serum and decreased the thymus index. TNF-α is a key cell signaling protein in the inflammatory response and induces apoptosis in diverse cell types (28). The observations here indicate that immunological stress may stimulate an inflammatory response and inhibit thymus development which subsequently leads to a decline in broiler immune function. Based on the preceding results, we speculate that the primary cause of LPS-mediated growth inhibition of broilers involves enhancement of the synthesis and secretion of stress hormones. These hormones may stimulate signal molecule receptors that are implicated in appetite control and growth which promote increased catabolism, anorexia, and growth inhibition. Alternatively, pro-inflammatory cytokines that are produced as a consequence of immunological stress may repartition nutrients and increase the catabolic activities that are associated with broiler growth.

The hypothalamus plays a key role in the regulation of feeding (29). The hypothalamic nuclei involved in feeding control include the arcuate nucleus (ARC), paraventricular nucleus, ventromedial nucleus, dorsomedial nucleus, and lateral hypothalamic area. These loci form a regulatory network for feeding activities and maintenance of energy metabolism (30, 31). ARC is the most critical nucleus for feeding regulation (32). Two principal neuronal populations that modulate appetite conversely exist in the ARC: AgRP neurons which co-express NPY, and POMC neurons. Activation of POMC decreases daily feed intake, whereas stimulation of AgRP/NPY boosts feed intake (33–36). Here we found that the feed intake of broilers was reduced 72 h after the first LPS challenge and that the expression of POMC in the hypothalamus of these animals was increased. The expression of NPY also was elevated compared with the control group which is inconsistent with previous studies (37–40). We speculate that expression of the SOCS3 gene was enhanced in the hypothalamus of stressed broilers within 2 h of the primary LPS injection. The SOCS3 protein is the main inhibitor of leptin and insulin signaling which causes cellular leptin and insulin resistance and dampens energy production (41–43). This state of energy deficiency may increase NPY expression temporarily in the hypothalamus. Thus, our results indicate that POMC may be the key regulatory factor in LPS-induced anorexia which was reflected in the decreased feed intake of broilers 72 h after LPS administration. However, the effect of LPS on NPY expression requires further exploration.

The hypothalamic-pituitary-growth hormone axis plays a crucial role in the regulation of growth (44). The hypothalamus secretes GHRH and SST hormones which act on the pituitary gland with opposing effects to modulate growth hormone (GH) secretion: GHRH promotes GH secretion whereas SST inhibits GH secretion. GH enters the blood and binds to the liver growth hormone receptor to stimulate the synthesis and secretion of IGF-1 by liver cells. IGF-1 interacts with binding proteins on target cells and tissues and enhances the growth and development of skeletal muscle and the cardiovascular and cerebrovascular systems (45, 46). GHRH may be attenuated by a pronounced increase in SST concentrations or, conversely, SST may be potentiated by GHRH stimulation (47). Although stimulation of GHRH appeared to predominate compared with SST stimulation in response to LPS in this study, we found that body weight gain of broilers in the immune stress group was reduced at 24 h after the first LPS injection. Moreover, the decline of GHRH secretion in the hypothalamus was accompanied by a decrease of IGF-1 levels in serum 2 h after LPS challenge. However, the expression of SST in the hypothalamus of stressed broilers did not fluctuate. Therefore, the growth inhibition of stressed broilers partly may reflect inhibition of the activity of hypothalamic GHRH neurons which negatively regulate the hypothalamic-pituitary-growth hormone axis which in turn leads to the decrease of production performance. Thus, immune stress decreased GHRH secretion and increased POMC secretion which reduced feed intake and slowed the body weight gain of stressed broilers. However, the mechanism by which immune stress affects GHRH and POMC expression in stressed broilers needs further investigation.

The NF-κB and p38/MAPK signaling pathways drive the expression of inflammatory mediators and enzymes involved in the inflammatory response, including COX-2 (48, 49). COX-2 is a key enzyme in the epoxidase pathway of the arachidonic acid metabolic network and expression of COX-2 profoundly affects the production of PGs (12). Here, we observed that LPS administration activated the MAPK-NF-κB pathway though the TLR1 receptor and MyD88, and increased the secretion of COX-2 and mPGEs-1 in the hypothalamus of stressed animals. The enhancement of COX-2 expression preceded mPGEs-1 expression. Recent studies have revealed that COX-2 participates in the production of LPS-induced proinflammatory factors by activating microglia-related signaling pathways (50). We found here that COX-2 was released early and produced at the same time point as IL-1β and CD40 in the hypothalamus of immune-stressed broilers. Thus, COX-2 may participate in the production of IL-1β by activating microglia-related signaling pathways. The IL-1β cytokine induces PGE_2_ production *via* up-regulation of COX-2 and mPGEs-1 expression in several cell types (51). In addition, the function of PGE_2_ mainly is mediated by G protein-coupled receptors located in the cell membrane. EP receptors comprise four subtypes (EP1, EP2, EP3, and EP4) (52). Here, the EP4 gene was significantly up-regulated in stressed broilers whereas expression of EP1, EP2, and EP3 did not change significantly. Genome-wide association studies previously revealed that polymorphisms in the EP4 gene are associated with overexpression of EP4 and a more severe disease phenotype in patients with irritable bowel disease (53–55). Moreover, certain variants in the EP4 gene are associated with Crohn’s disease (56). These observations suggest that COX-2-PGE_2_-EP4 signaling also may participate in the pathogenesis of inflammation. These findings suggest that IL-1β induced by LPS regulates the key components of the PGE_2_ biosynthetic cascade, e.g., COX-2, mPGEs-1, and EP4, through the MAPK-NF-κB pathway. The combination of PGE_2_ and EP4 receptor in microglia may trigger a positive feedback loop that promotes the amplification of COX-2 effects during LPS challenge. Therefore, the decrease in growth performance of broilers under immune stress is associated strongly with COX-2 signaling mediated by the NF-κB/MAPK signaling pathway.

The immune and neuroendocrine systems are connected closely. Inflammatory cytokines induced by LPS directly contact microglia in the hypothalamus and promote production of increased concentrations of COX-2 by these cells. The level of PGE_2_ increases as a consequence and this PG diffuses to the feeding center to cause animal anorexia and inhibition of food intake (57–59). Analogously, other studies found that the application of COX-2 inhibitors alleviated the loss of appetite caused by LPS in poultry (10) which suggests that COX-2 may mediate the suppression of feed intake of animals under immune stress. Deletion of the gene for EP4 in microglial cells reduced microglial-POMC neuron contacts (60) and implicated PGE_2_ signaling *via* EP4 as an important regulator of microglia-POMC neuron interactions. In addition, accumulating evidence has substantiated that IL-1 affected hypothalamic peptide release were mediated *via* COX stimulation of PGE synthesis. IL-1-induced stimulation of GHRH and SST are antagonized by indomethacin which inhibits PG synthesis *via* the COX pathway (61). In view of these observations, we speculate that LPS-induced growth inhibition may be mediated by the COX-2-mPGEs-1-PGE_2_ axis in the hypothalamus: PGE_2_ binding to EP4 receptors *via* appetite and growth regulatory peptides thereby may affect growth and development in stressed broilers.

The molecular mechanism of stress-activated COX-2 signaling in growth regulation of broiler chickens was probed further by establishing a celecoxib pretreatment group. Celecoxib is a new, non-steroidal anti-inflammatory drug that selectively inhibits COX-2 enzymatic activity and the subsequent pro-inflammatory factor, PGE_2_ (62). Clinical studies demonstrated that celecoxib passes through the blood-brain barrier (63). Our results here indicate that pretreatment with celecoxib increased the body weight gain of stressed broilers, and also resolved inflammation in the serum and hypothalamus. Moreover, CD40 levels also were reduced which suggests that microglia displayed a less activated phenotype (64). In addition, celecoxib significantly down-regulated the expression of CX3CL1, CCL4, IRF7, LY96, MAP3K8 and TLR1B in the MAPK-NF-κB signaling pathway. These observations verify our previous results in this study which indicated that COX-2 may act as an important mediator in the activation of the AMPK-NF-κB signaling pathway *via* the EP4 receptor. Furthermore, our finding that inhibition of COX-2 activity elevated GHRH expression in the hypothalamus of stressed animals without affecting SST expression is consistent with our previous inference that COX-2 signaling plays an important role in LPS-induced growth inhibition by binding to EP4 receptors on GHRH neurons to moderate GHRH expression. It is important to note that we observed that inhibition of COX-2 activity increased NPY expression but exerted no detectable effect on POMC. Combined with previous findings, these data imply that LPS-induced anorexia may derive from the concerted action of NPY and POMC. COX-2 signaling may inhibit growth in stressed broilers by binding to EP4 receptors on NPY neurons, in which case the activity of POMC neurons may not be impacted by the COX-2 pathway. LPS causes inflammation systematically and the NF-кB signaling pathway interacts directly with POMC to promote the transcription and expression of the POMC gene (65). Therefore, the decline in food intake among stressed broilers caused by the up-regulated expression of POMC may be mediated by the NF-кB signal pathway in our study.

## 5 Conclusion

**Figure 10:**
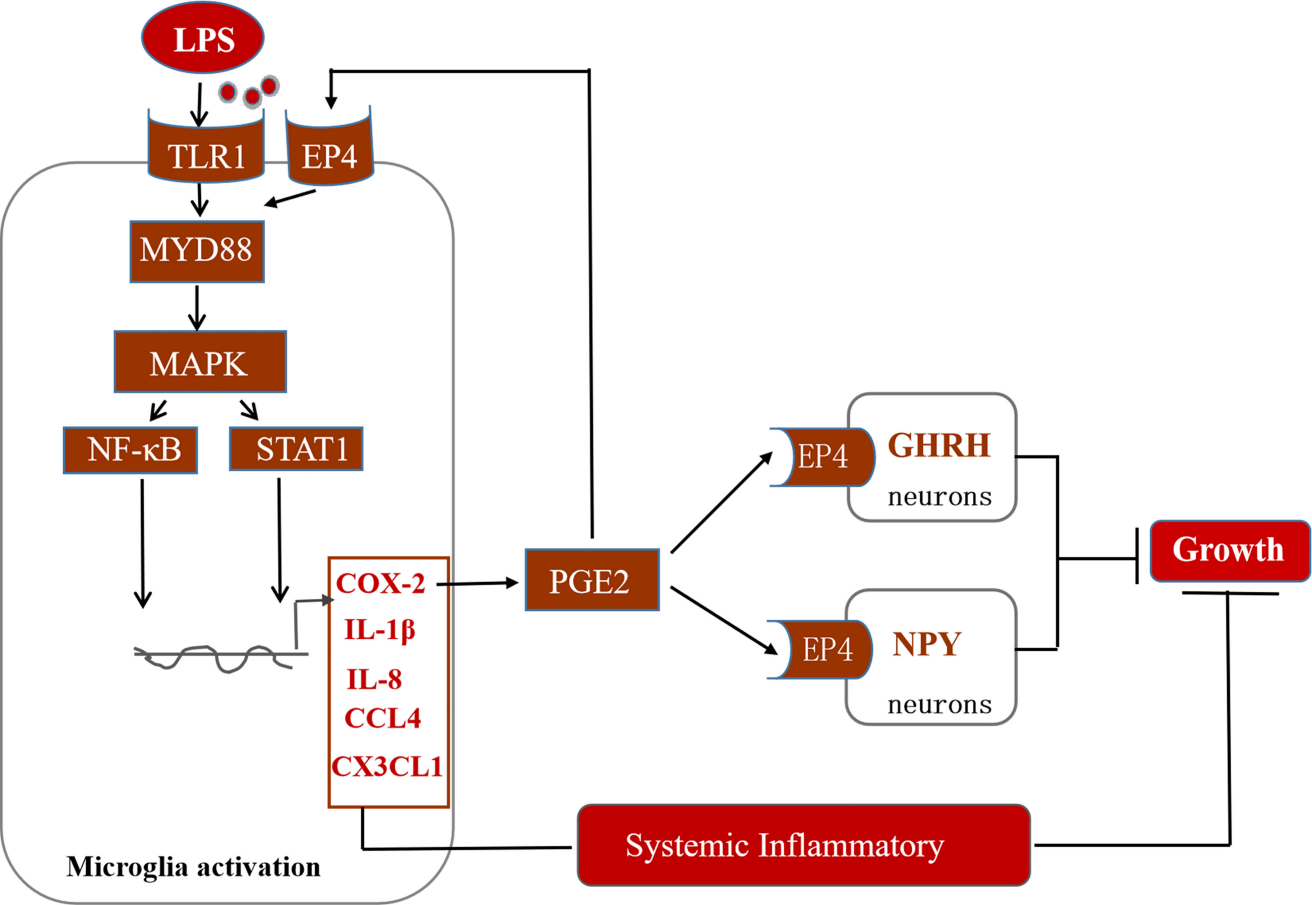
Potential mechanisms of action of hypothalamic COX-2 in the regulation of growth in stressed broilers.

In conclusion, this study suggests that immune stress regulates the MAPK-NF-κB signaling pathway and drives the transcription of inflammatory factors such as COX-2 (**Figure 10**). COX-2-PGE2 signaling induces the secretion of related inflammatory factors *via* the EP4 receptor on microglia. Moreover, PGE2-EP4 also may cascade with the appetite regulatory peptide NPY and the growth regulatory factor GHRH to dampen the concentration of IGF-1 in the serum of stressed broilers. We propose that this relay in turn decreases feed intake and body weight gain and leads to the suppression of growth of immune stressed broilers.

## Data availability statement

The datasets presented in this study can be found in online repositories. The names of the repository/repositories and accession number (s) can be found below: PRJNA940969 (SRA).

## Ethics statement

The animal study was reviewed and approved by Animal Care and Use Committee of Henan University of Science and Technology.

## Author contributions

Investigation, KL, HT, and XH. Methodology, KL, XH, and HT. Software, KL, HT, and WZ. Formal analysis, KL, YZ, DB, and WZ. Data curation, KL, HT, YQL, and YHL. Writing the original draft, KL, WZ, and DB. Reviewing and editing, DB, KI, WZ and BZ. Supervision, YM and project administration, YM. All authors contributed to the article and approved the submitted version.
