# Supplementary material for: Lipopolysaccharide-induced immune stress negatively regulates broiler chicken growth via the COX-2-PGE2-EP4 signaling pathway
Source: Front Immunol. 2023 May 3;14:1193798. doi: 10.3389/fimmu.2023.1193798 (PMC10189118; doi:10.3389/fimmu.2023.1193798)
Supplement: Supplementary file 1 [file Table_1.docx]

Supplementary Material

**Supplementary Tables**

Table S1. LPS-induced immune stress regulates the MAPK-NF-κB signaling pathway.

| ID | Gene names | | | Log2(Fold Change) | | *P* value | Style | |
| --- | --- | --- | --- | --- | --- | --- | --- | --- |
| ENSGALG00000043167 | | TNFRSF11B | | | 2.691642412 | 3.77E-88 | | up |
| ENSGALG00000027864 | | NFKBIA | 2.282333695 | | | 1.21E-62 | | up |
| ENSGALG00000039741 | | TNFRSF8 | 3.722503769 | | | 6.32E-47 | | up |
| ENSGALG00000003863 | | P2RX7 | 3.420772143 | | | 9.36E-36 | | up |
| ENSGALG00000006106 | | TNFRSF6B | 2.850149682 | | | 2.14E-22 | | up |
| ENSGALG00000015346 | | NFKBIZ | 1.86049616 | | | 6.05E-21 | | up |
| ENSGALG00000034478 | | CCL4 | 5.528368461 | | | 1.79E-15 | | up |
| ENSGALG00000030291 | | IRF9 | 2.062353415 | | | 1.93E-14 | | up |
| ENSGALG00000026663 | | CX3CL1 | 4.642900186 | | | 6.46E-13 | | up |
| ENSGALG00000014297 | | IRF7 | 2.157196177 | | | 1.56E-12 | | up |
| ENSGALG00000026098 | | IL8 | 5.044893362 | | | 2.11E-11 | | up |
| ENSGALG00000043535 | | RIPK2 | 1.179246837 | | | 1.51E-10 | | up |
| ENSGALG00000028256 | | CCL19 | 2.871768748 | | | 5.02E-09 | | up |
| ENSGALG00000032701 | | LY96 | 1.930779539 | | | 5.74E-06 | | up |
| ENSGALG00000036627 | | CXCR1 | 2.465491803 | | | 6.95E-06 | | up |
| ENSGALG00000028037 | | FOS | 1.319804349 | | | 3.28E-04 | | up |
| ENSGALG00000043603 | | CCL5 | 3.992844769 | | | 3.42E-02 | | up |

Transcript levels of differential genes in the hypothalamus from the LPS groups compared to the saline group 2 h after the first injection. [Log2(Fold Change)] ≥ 1 and p<0.05 means significant difference between groups.

**Table S2. COX-2 inhibitor celecoxib attenuated LPS-induced growth inhibition and inflammation.**

| ID | Gene names | Log2(Fold Change) | *P* value | Style |
| --- | --- | --- | --- | --- |
| ENSGALG00000003863 | P2RX7 | -2.859196998 | 3.74E-28 | down |
| ENSGALG00000043167 | TNFRSF11B | -1.833429375 | 8.55E-23 | down |
| ENSGALG00000006106 | TNFRSF6B | -2.603580791 | 3.60E-21 | down |
| ENSGALG00000030291 | IRF9 | -2.229611949 | 2.32E-15 | down |
| ENSGALG00000010628 | ACSLI | -1.166162198 | 9.73E-15 | down |
| ENSGALG00000039741 | TNFRSF8 | -2.117438845 | 6.92E-13 | down |
| ENSGALG00000027093 | TLR1B | -1.605178896 | 2.58E-12 | down |
| ENSGALG00000003217 | LITAF | -2.472675206 | 4.04E-12 | down |
| ENSGALG00000005857 | ALOX5 | -1.255446484 | 2.40E-11 | down |
| ENSGALG00000050893 | CCR2 | -1.578132591 | 1.53E-10 | down |
| ENSGALG00000037989 | IL10RB | -1.200751833 | 7.34E-10 | down |
| ENSGALG00000043535 | RIPK2 | -1.139052869 | 4.48E-09 | down |
| ENSGALG00000027786 | SOCS3 | -2.600535727 | 1.34E-08 | down |
| ENSGALG00000028256 | CCL19 | -2.76570785 | 2.24E-08 | down |
| ENSGALG00000040504 | GGT5 | -1.491378356 | 3.05E-08 | down |
| ENSGALG00000026663 | CX3CLI | -3.088377753 | 3.74E-07 | down |
| ENSGALG00000020316 | IL13RA2 | -2.525327133 | 5.29E-06 | down |
| ENSGALG00000036627 | CXCR1 | -2.459912541 | 6.78E-06 | down |
| ENSGALG00000006351 | FAS | -1.074568426 | 7.67E-06 | down |
| ENSGALG00000007015 | CD40 | -1.472912992 | 7.69E-06 | down |
| ENSGALG00000034478 | CCL4 | -2.706719132 | 1.07E-05 | down |
| ENSGALG00000046326 | IL1R2 | -3.130162887 | 1.91E-05 | down |
| ENSGALG00000041192 | IFIH1 | -1.55916736 | 2.15E-05 | down |
| ENSGALG00000011418 | CCR6 | -1.411509286 | 7.77E-05 | down |
| ENSGALG00000007158 | SOCSI | -2.05656279 | 0.000113 | down |
| ENSGALG00000032701 | LY96 | -1.54994773 | 0.000242 | down |
| ENSGALG00000012396 | PTGER2 | -1.766117162 | 0.000598 | down |
| ENSGALG00000014297 | IRF7 | -1.125533797 | 0.000868 | down |
| ENSGALG00000007356 | MAP3K8 | -1.227953428 | 0.00102 | down |
| ENSGALG00000016785 | IL1RL1 | -1.06533036 | 0.001748 | down |
| ENSGALG00000013861 | TNFAIP3 | -1.11469088 | 0.004283 | down |
| ENSGALG00000010926 | SPP1 | -3.224626432 | 0.033009 | down |
| ENSGALG00000032260 | IL10RA | -1.084615796 | 0.049365 | down |

Transcript levels of differential genes in the hypothalamus from the LPS and celecoxib groups compared to the LPS group 2 h after the first injection. [Log2(Fold Change)] ≥ 1 and p<0.05 means significant difference between groups.
